# Tinea Capitis at Ibn Sina Hospital in Rabat, Morocco: Epidemiological and Etiological Study Over 25 Years (From 1997 to 2021)

**DOI:** 10.7759/cureus.57885

**Published:** 2024-04-09

**Authors:** Fataou Saley Younoussa, Mehdi Elouadani, Mohammed Lyagoubi, Sarra Aoufi

**Affiliations:** 1 Central Laboratory of Parasitology and Mycology, Faculty of Medicine and Pharmacy, Ibn Sina Hospital, Mohammed V University, Rabat, MAR

**Keywords:** morocco, microsporum canis, trichophyton violaceum, tinea capitis, epidemiology

## Abstract

Introduction/Objective

In Morocco, tinea capitis is a common reason for consultation, especially in children. Our study aimed to determine the epidemiology of tinea capitis in the Central Laboratory of Parasitology and Mycology at the Ibn Sina University Hospital Center (UHC) in Rabat.

Materials and methods

This is a retrospective study conducted over 25 years (from 1997 to 2021). It included 247 patients with lesions suggestive of tinea capitis, who underwent a mycological examination.

Results

Among 594 requests for the diagnosis of tinea capitis, 247 cases were positive. A clear predominance of children (86.23%; n=213) as compared to adults (13.77%; n=34) was observed. The sex ratio (M/F) was 1.77. Hair parasitism was mainly dominated by the pure endothrix type 54.47% (n=122). The two most frequently isolated species were *Trichophyton violaceum* (51.24%; n=125) and *Microsporum canis* (36.06%; n=88). In children, these two species represented 47.42% (n=101) and 41.31% (n=88), respectively. In adults, *Trichophyton violaceum* was the predominant species, accounting for 77.42% (n=24); in females, it was 76.41% (n=68); and *Microsporum canis* was predominant in males (50.32%; n=78).

Conclusion

The epidemiology of this condition is in a constant state of flux, influenced by various lifestyle factors. Our research unveiled a notable upward trend in zoophilic species over the 25-year study period, while conversely, anthropophilic species demonstrated a discernible decline.

## Introduction

Tinea capitis (TC) is a superficial mycosis caused by dermatophytes, which are keratinophilic filamentous fungi that mainly affect school children. These microscopic fungi belong to the phylum Ascomycota. Most of them are mitosporic, with unknown sexual states, and the names of their anamorphs in the genera *Microsporum*, *Trichophyton*, and *Epidermophyton* are commonly used in the medical literature. Only the first two genera have species capable of invading terminal hair and, therefore, causing scalp ringworm [1]. Tinea capitis accounts for 25-30% of all fungal infections [2]. The geographic origin and lifestyle factors, particularly the rise in pet adoptions, migratory flows, and personal hygiene, contribute to the constantly changing epidemiology of this pathology. In Morocco, tinea capitis is a common reason for consultation, especially in children. This study aims to determine the epidemiological and etiological profiles of tinea capitis diagnosed at the Central Laboratory of Parasitology and Mycology of the Ibn Sina University Hospital Center (UHC) in Rabat.

## Materials and methods

This is a retrospective study conducted over 25 years (from January 1997 to December 2021). It involved 247 patients with suggestive lesions of tinea capitis (small or large patches of alopecia, inflammatory or not), who underwent a mycological examination at the Central Laboratory of Parasitology and Mycology of the Ibn Sina UHC in Rabat. Data were collected from the laboratory bench registers. For each patient, the following parameters were recorded: age, sex, geographical origin, contact with animals, microscopic examination, and culture results.

Data entry was carried out using Microsoft Excel 2019 software (Microsoft Corporation, Redmond, WA, USA), and statistical analysis was performed using SPSS v23.0 software (IBM Corp., Armonk, NY, USA). Categorical variables were compared using the chi-square test while quantitative variables were analyzed using the Mann-Whitney U test. Results were considered significant for all statistical tests when P < 0.01.

The sampling was performed before the administration of any antifungal treatment; if not, a therapeutic window of 15 days for topical antifungals and 2-3 months for systemic antifungals was respected.

We included patients of all ages referred for mycological scalp sampling during the study period who presented a positive result on mycological microscopic examination and/or culture. We excluded those who had received antifungal treatment.

The microscopic examination (10X and 40X) was performed after clearing with potassium hydroxide (KOH 30%), followed by a light microscopic reading at objectives 10 and 40.

The samples were cultured on different Sabouraud media: Sabouraud Chloramphenicol agar and Sabouraud Chloramphenicol Actidion agar, and then incubated at 27°C. They were checked once a week for three weeks. Cultures were considered negative if no growth was observed after one month.

The identification of the causative agent was based on the time of growth, the macroscopic appearance of colonies on both the front and back, the development and potential diffusion of pigments, as well as their microscopic appearance in lactophenol blue stain, which included the observation of conidiation and/or ornamentations. Subcultures on media that stimulate conidiation and, when necessary, for some species, the production of pigments was performed.

## Results

Demographic data

**Figure 1:**
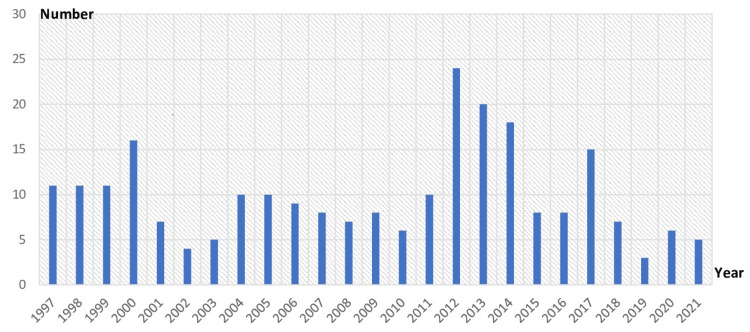
Annual number of cases of tinea capitis

Of 594 requests, 247 cases were positive, resulting in an overall prevalence of 41.58%. Figure 1 shows the annual tinea capitis cases in our study.

**Figure 2:**
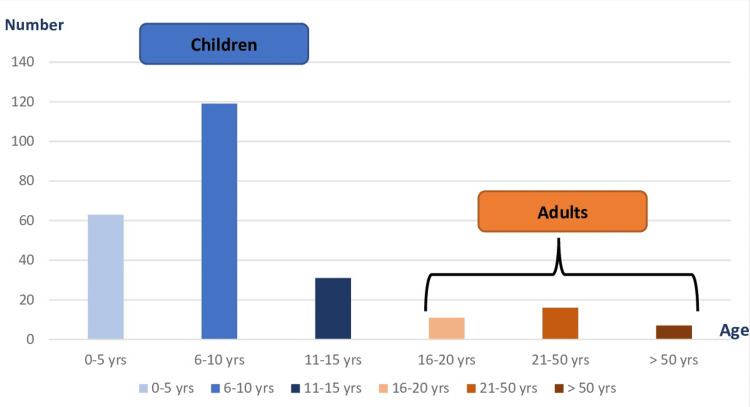
Distribution of tinea capitis according to age

A clear predominance of children (86.23%, n=213) compared to adults (13.77%, n=34) was observed. The most affected age group was that between 6 and 10 years as shown in Figure 2.

The sex ratio (M/F) was 1.77. Among children, males were the most affected with a rate of 69.48% (n=148). However, in the adult group, females were the most affected, accounting for 70.59% (n=24) (Table 1).

**Table 1:** Distribution of patients according to age and sex

	Adult			Children		
Sex	Male	Female	Total	Male	Female	Total
Number of cases	10	24	34	148	65	213
%	29.41	70.59	100	69.48	30.52	100

Clinical and mycological data

The type of clinical lesion was determined in 135 patients. The dominant clinical presentation was large patches of alopecia (60 %, n=81). Of 247 patients, 224 had a positive microscopic examination. Hair parasitism was dominated by the pure endothrix type (54.47%, n=122), followed by the microsporic endoectothrix (23.66%, n=53). However, in 13.84% of the cases (n=31), the type of hair parasitism was not determined (Table 2).

**Table 2:** Distribution according to the type of tinea capitis and the type of hair parasitism

Lesion type	Cases	%
Inflammatory lesions – Kerion	15	11.11
Scaly lesions	15	11.11
Hair loss	2	1.48
Small alopecic patches	22	16.3
Large alopecic patches	81	60
Total	135	100
Microscopic examination	Cases	%
Pure endothrix parasitism	122	54.47
Microsporic endoectothrix parasitism	53	23.66
Megasporic endoectothrix parasitism	8	3.57
Favic parasitism	10	4.46
Mycelial filaments + spores	31	13.84
Total	224	100

The culture was positive in 244 cases. Three cases had negative cultures and positive microscopic examinations. Anthropophilic species represented 60.24% (n=147) while zoophilic species represented 39.76% (n=97).

**Figure 3:**
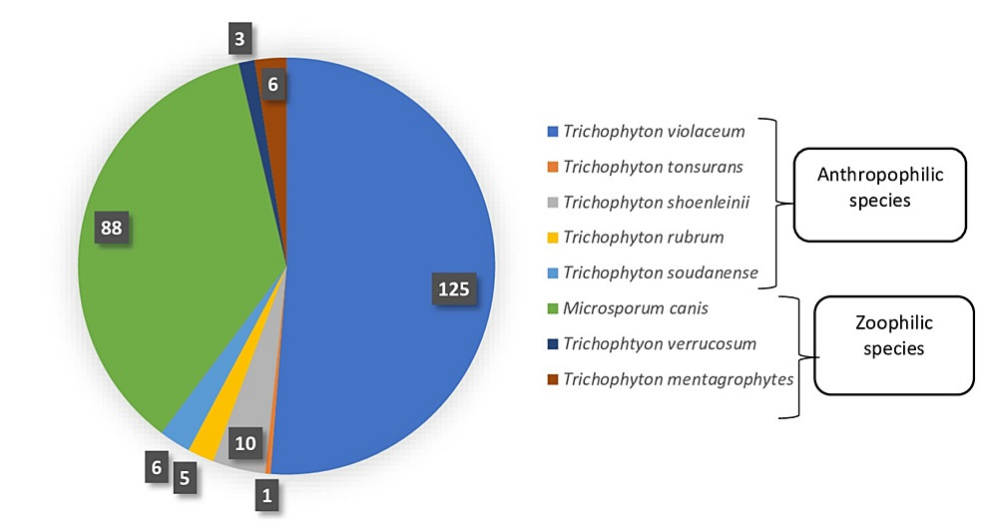
Number of cases by species isolated in culture

The most isolated species were *Trichophyton violaceum* (51.24%, n=125), and *Microsporum canis* (36.06%, n=88). In addition, we isolated *Trichophyton soudanense* in six patients (2.46%) and *Trichophyton tonsurans* in one patient (0.40%) (Figure 3). 

According to the age and sex of our patients, it appears that in children, *Trichophyton violaceum* and *Microsporum canis* were the most frequently isolated species with 47.42% (n=101) and 41.31% (n=88), respectively, statistically significant (p < 0.01). In adults, *Trichophyton violaceum *was the predominant species, accounting for 77.42% (n=24).

Similarly, we found a significant difference in the frequency of species isolated between sexes, with a predominance of *Trichophyton violaceum* in females (76.41%, n=68), compared to males (36.77%, n=57), statistically significant (p < 0.01). On the other hand, *Microsporum canis* predominated in males (50.32%, n=78), compared to females (11.24%, n=10). In female adults, *Trichophyton violaceum* was the only species isolated.

**Figure 4:**
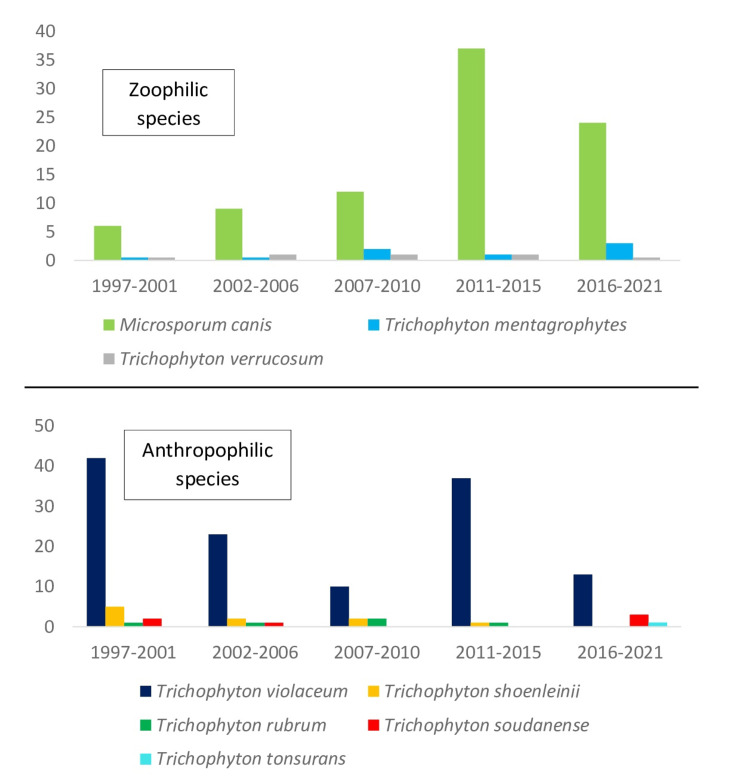
Evolution of species number by year

A progressive increase in zoophilic species (*Microsporum canis*, *Trichophyton mentagrophytes*, and *Trichophyton verrucosum*) was observed over the course of the 25-year study while anthropophilic species (*Trichophyton violaceum *and *Trichophyton shoenleinii*) tended to decrease (Figure 4).

## Discussion

Tinea capitis (TC) is a widespread public health concern in developing countries. It can heal spontaneously at puberty or become superinfected and progress to more severe forms [3].

Mycological examination is essential for the diagnosis of tinea capitis. It involves microscopic examination and culture. The positivity of microscopic examination allows immediate therapeutic management and limits the risk of contamination of the environment [4-6].

The prevalence identified in our study, at 41.58%, underscores the persistent status of TC as a significant national public health concern. Furthermore, this prevalence closely aligns with the findings of a retrospective study conducted in Morocco, which reported a rate of 43.85% [7]. Similar findings have also been documented in studies from Algeria, Senegal, and Tunisia, further affirming the consistency of these concerning statistics [8,9].

According to the World Health Organization (WHO), TC ranks as the second most common childhood dermatological disease in developing countries [2]. It is primarily observed in individuals post-puberty, with an occurrence rate ranging from 3% to 5% [10,11]. Furthermore, TC is not uncommon among adults.

Children were more affected than adults in our study, which is in agreement with what has been reported by several authors [12-14]. The low prevalence in adults may be related to the fungistatic properties of the triglycerides constituting the sebum and sex hormones, which explains the spontaneous recovery of patients at puberty [15].

Our sex ratio is comparable to that reported in another Moroccan study (1.14) [16] and in an Algerian study (1.8) [14]. In children, males were the most affected, however, females were the most affected in adults, which agrees with a study conducted in France [13].

The disparity in distribution between genders can be attributed to the substantial hair growth in girls, acting as a barrier that impedes the spores responsible for infection from reaching the scalp, in contrast to boys with shorter hair. Moreover, it could be linked to cultural and hormonal factors. Indeed, girls are more quickly sensitized to hygiene and puberty.

Clinically, large patches of alopecia are the most frequent clinical manifestation in our patients. The same finding was reported in an Algerian study [8].

Microscopic examination can confirm the mycological origin of the infection and specify the type of hair parasitism that points to a specific type of fungus. The dominant type of hair parasitism in our series was pure endothrix, followed by the microsporic endoectothrix. However, Algerian authors have reported a predominance of the microsporic endoectothrix type, followed by the microid endoectothrix type [8]. The geographical origin as well as the lifestyle could explain this disparity.

In our study, the identification of positive cultures revealed a predominance of anthropophilic species, which agrees with a study carried out in France [13].

In Morocco, the species responsible for TC have not changed since the studies conducted in 1992, 2010, and 2012 [12,17,18]. They were mainly due to *Trichophyton violaceum*, *Microsporum canis, *and *Trichophyton schoenleinii*.

In our study, the two most frequently isolated species were *Trichophyton violaceum* and *Microsporum canis*. These results agree with those of several studies in Morocco and Algeria [12,14,19] and differ from those reported by studies in Senegal and Gabon [7,20].

*Trichophyton violaceum* and *Microsporum canis* were found in almost the same proportions in children while a study conducted in Algeria has shown that *Trichophyton violaceum* is more frequent in children than *Microsporum canis* [14]. Concerning adults, in our study, *Trichophyton violaceum* was the predominant species in adults. Furthermore, *Trichophyton violaceum* predominated in females, whereas *Microsporum canis* was more prevalent in males. In an Algerian study, *Trichophyton violaceum* and *Microsporum canis* displayed an equal prevalence among females while *Microsporum canis* emerged as the predominant strain among males [14].

*Trichophyton schoenleinii*, a species classically found in North Africa, was isolated in 4.1% of cases (n=10). Some studies in Rabat reported a similar rate [12,16] while another reported a single case [19]. This particular species has become exceptionally rare, as it has not been isolated in our unit since 2015.

We isolated *Trichophyton soudanense* and *Trichophyton tonsurans* only in foreign patients. These are anthropophilic agents responsible for trichophytic tinea with small alopecia patches. Their geographical distribution is limited to central Africa for *Trichophyton soudanense*, and North America for *Trichophyton tonsurans* [21]. A Mauritanian study reported a case of *Trichophyton tonsurans* [22] while in France, both species are increasingly isolated [13]. The emergence of these species would be due to the important flow of immigrants from sub-Saharan Africa [23].

The epidemiology of TC is subject to continuous fluctuation, underscoring the need for biologists to maintain surveillance regarding the emergence of agents that were theoretically confined to specific geographical areas and might exhibit resistance to antifungal treatments, such as *Trichophyton tonsurans*, which has a decreased sensitivity to griseofulvin, the main antifungal agent used to treat TC [13].

It should also be noted that the taxonomy of dermatophytes is currently being reconsidered based on molecular phylogeny. In fact, molecular species definitions do not always coincide with existing concepts, which are guided by ecological and clinical principles.

Phylogenetic analysis using different molecular markers has clarified much of the dermatophyte taxonomy [24]. For example, sequenced strains of *Trichophyton rubrum* and *Trichophyton violaceum* shared high colinearity at the nucleotide level, with 99.0% identity [25].

The etiological distribution of TC depends on several factors (environment, lifestyle, level of hygiene, among others). We have noted a recrudescence of zoophilic species (*Microsporum canis*, *Trichophyton mentagrophytes*, and *Trichophyton verrucosum*) over the years of the study, contrary to anthropophilic species (for instance, *Trichophyton violaceum *and *Trichophyton shoenleinii*, to mention a few), which tend to decrease. This trend of zoophilic species is also reported by a 60-year study from China [26]. The increasing popularity of pets (dogs and cats) explains the increase in the frequency of certain zoophilic species, in particular *Microsporum canis*.

The results of our study must be interpreted while taking its limitations into account. Indeed, like all studies, ours has limitations. It is a retrospective study, restricted to data contained within the laboratory bench registers. Despite spanning a relatively long period, it remains a single-center study. Regarding laboratory diagnosis, molecular identification techniques were not carried out, and species identification was based on phenotypic characteristics.

## Conclusions

Tinea capitis remains a frequent reason for consultation in Morocco, especially in school children. Its epidemiology is constantly changing, depending on lifestyle factors, especially the increase in pet adoptions, migratory flows, and personal hygiene.

A mycological examination is the cornerstone for the management and differential diagnosis of other non-mycological scalp diseases. Thus, it facilitates treatment orientation and confirms the diagnosis.
